# Perioperative Neurocognitive Disorders: A Narrative Review of Pathophysiology, Prevention, and Management Strategies

**DOI:** 10.3390/jcm15031253

**Published:** 2026-02-04

**Authors:** Daniele Salvatore Paternò, Luigi La Via, Antonio Putaggio, Angela Piccolo, Giuseppe Scibilia, Mario Lentini, Antonino Maniaci, Fabrizio Luca, Emilia Concetta Lo Giudice, Massimiliano Sorbello

**Affiliations:** 1 Department of Anesthesia and Intensive Care, “Giovanni Paolo II” Hospital, 97100 Ragusa, Italy; paternomd@icloud.com (D.S.P.); dr.antonioputaggio@gmail.com (A.P.); angelamariapiccolo3@gmail.com (A.P.); 2 Department of General Surgery and Medical-Surgical Specialties, University of Catania, 95123 Catania, Italy; luigilavia7@gmail.com; 3 Department of Obstetrics and Gynecology, School of Medicine and Surgery, “Kore” University, 94100 Enna, Italy; giuseppe.scibilia@unikore.it; 4 Department of Ear Nose and Throat, School of Medicine and Surgery, “Kore” University, 94100 Enna, Italy; mario.lentini@unikore.it (M.L.); antonino.maniaci@unikore.it (A.M.); 5 Department of Surgery, School of Medicine and Surgery, “Kore” University, 94100 Enna, Italy; fabrizio.luca@unikore.it; 6 Department of Anesthesia and Intensive Care, School of Medicine and Surgery, “Kore” University, 94100 Enna, Italy; emilia.logiudice@unikore.it

**Keywords:** postoperative delirium, postoperative cognitive dysfunction, neuroinflammation, risk stratification, multicomponent interventions, preventive strategies, elderly surgical patients, cognitive impairment

## Abstract

**Background/Objectives**: Perioperative neurocognitive disorders (PNDs), including delirium and postoperative cognitive dysfunction, affect 10–50% of elderly surgical patients and are associated with increased morbidity and mortality, as well as substantial healthcare costs. Despite their clinical significance, the underlying mechanisms remain incompletely understood and effective interventions are limited. This narrative review synthesizes current evidence on the pathophysiology, risk factors, and management strategies for PNDs. **Methods:** We conducted a comprehensive literature review of peer-reviewed publications addressing PND epidemiology, mechanisms, assessment, and interventions. Key databases were searched for studies published through 2025, with emphasis on systematic reviews, meta-analyses, and landmark clinical trials. **Results:** PND represents a spectrum of cognitive impairments with multifactorial etiology involving neuroinflammation, neurotransmitter imbalances, and blood–brain barrier dysfunction. Advanced age, pre-existing cognitive impairment, and surgical factors constitute major risk domains. Validated assessment tools including the Confusion Assessment Method (CAM) and 4AT enable systematic detection. Multicomponent non-pharmacological interventions demonstrate 30–40% delirium reduction, while pharmacological prevention shows limited efficacy. Emerging evidence links perioperative delirium to accelerated long-term cognitive decline and increased dementia risk. **Conclusions:** PND represents a significant public health challenge requiring systematic attention in aging surgical populations. Evidence-based multicomponent interventions should be integrated into routine perioperative care pathways. Future research must elucidate mechanistic pathways linking acute delirium to chronic cognitive impairment and develop targeted therapies to preserve cognitive health in surgical populations.

## 1. Introduction

Perioperative neurocognitive disorders (PNDs) encompass a range of cognitive impairments that develop in connection with surgery and anesthesia. As surgical volumes continue to rise globally—exceeding 300 million procedures annually [1]—and populations age [2], these disorders have become an increasingly pressing public health challenge. PND includes both acute conditions, such as postoperative delirium (POD), and more persistent cognitive dysfunction that can last weeks to months after surgery [3]. The impact of PND reaches well beyond the operating room and immediate recovery period. Elderly patients, who now represent a growing segment of surgical populations [4], are particularly vulnerable. Depending on the procedure and individual patient factors, postoperative delirium affects between 10% and 50% of older surgical patients, with incidence rates climbing toward 50% in emergency operations and complex cardiovascular surgeries [3]. Beyond the human cost, the economic burden is staggering: in the United States alone, delirium-related healthcare expenditures exceed USD 164 billion each year [5]. The consequences of perioperative neurocognitive disorders extend into patients’ long-term futures. Postoperative delirium has been linked not only to increased mortality and prolonged hospital stays but also to higher rates of institutionalization and lasting functional decline [6,7]. Perhaps most troubling are recent findings from longitudinal studies suggesting that delirium may accelerate cognitive deterioration and raise the risk of dementia [8,9]. This emerging evidence points to a potential mechanistic connection between acute perioperative cognitive disturbances and chronic neurodegenerative processes—a link that carries profound implications for patient care and outcomes. Despite their clinical importance, significant gaps persist in our understanding of PNDs. The underlying pathophysiology remains incompletely characterized, and effective management strategies are limited. Detection poses a major challenge: studies indicate that delirium goes unrecognized in up to 60% of cases during routine clinical practice [10,11]. While multicomponent non-pharmacological interventions have shown promise in preventing delirium [12,13], their implementation across healthcare settings remains inconsistent. Meanwhile, pharmacological approaches to prevention and treatment have yielded disappointing results, and no medications are currently approved specifically for managing delirium. Recognition that cognitive changes can follow surgery and anesthesia is not new. Bedford’s landmark observations in 1955 first drew attention to “adverse cerebral effects of anaesthesia on old people” [14], with subsequent reports in 1959 further documenting these concerns [15]. However, it was the International Study of Postoperative Cognitive Dysfunction (ISPOCD) in 1998 that provided the first large-scale, systematic examination of the phenomenon [16]. This pivotal work established postoperative cognitive dysfunction as a distinct clinical entity deserving focused investigation. As populations continue to age and surgical volumes expand [17], addressing PND has taken on new urgency. Recent years have brought important progress: standardized nomenclature [18], deeper insights into pathophysiology, and the development of evidence-based prevention and management strategies. Yet translating these research advances into routine clinical practice remains a formidable challenge. This narrative review synthesizes current evidence on the pathophysiology, risk stratification, prevention, and management of perioperative neurocognitive disorders. We examine how nomenclature and classification systems have evolved, explore the mechanisms underlying PND development, review validated assessment tools, and critically evaluate both pharmacological and non-pharmacological interventions. Finally, we identify key knowledge gaps and outline future research directions needed to optimize cognitive outcomes for surgical patients.

## 2. Literature Search and Selection Strategy

This narrative review synthesizes current knowledge on perioperative neurocognitive disorders through a comprehensive literature search across PubMed/MEDLINE, Embase, Cochrane Library, and Web of Science from inception through January 2025. Our search strategy employed combinations of key terms including “perioperative neurocognitive disorder,” “postoperative delirium,” “postoperative cognitive dysfunction,” “POCD,” “PND,” “anesthesia,” and “surgery,” combined with terms addressing epidemiology, pathophysiology, prevention, and management.

Given the narrative nature of this review, we prioritized rather than systematically restricted our literature selection. We emphasized systematic reviews and meta-analyses, randomized controlled trials, large prospective cohort studies, clinical practice guidelines from major professional societies, and landmark studies that established foundational concepts. To ensure currency, we prioritized studies published between 2020 and 2025 while retaining earlier foundational work such as the ISPOCD studies and Hospital Elder Life Program development that remain highly cited and methodologically influential. Historical papers by Bedford from the 1950s were included to provide context for the field’s evolution. We favored studies with external validation, high citation impact, and evidence of replication, while including recent work that advances or challenges established concepts.

We sought geographic and population diversity, incorporating studies from multiple countries and healthcare systems representing diverse surgical populations including cardiac, orthopedic, general surgery, and ICU patients. Special attention was given to the 2018 nomenclature consensus statement, the 2024 European Society of Anaesthesiology and Intensive Care Medicine guideline update, large-scale individual patient data meta-analyses, recent mechanistic studies linking neuroinflammation to clinical outcomes, and emerging evidence on the delirium-dementia relationship.

We manually searched reference lists of key systematic reviews and guidelines and monitored clinical journals through citation alerts to capture very recent publications. The final reference list balances foundational evidence with contemporary research: of 78 total references, 23 (29.5%) were published in the past 5 years, 41 (52.6%) within the past 10 years, and 64 (82%) within the past 15 years, ensuring this review reflects both established knowledge and cutting-edge developments in perioperative neurocognitive disorders.

## 3. Classification and Nomenclature

### 3.1. Historical Evolution of Terminology

The terminology surrounding cognitive changes associated with surgery and anesthesia has evolved considerably over the past seven decades. Early descriptions by Bedford in the 1950s referred broadly to “adverse cerebral effects” and “confusional states” in elderly surgical patients [14,15], without clearly distinguishing between acute delirium and more prolonged cognitive changes. Throughout subsequent decades, various terms were employed inconsistently across research studies and clinical settings—including “postoperative confusion,” “acute brain failure,” “postoperative psychosis,” and “ICU syndrome”—creating confusion in the literature and hampering meaningful cross-study comparisons. The term “postoperative cognitive dysfunction” (POCD) gained prominence following the landmark ISPOCD studies in the late 1990s [16], which employed standardized neuropsychological testing to define cognitive decline. However, POCD itself remained poorly defined in terms of specific diagnostic criteria, duration of persistence, and clinical significance. Different research groups employed varying operational definitions, making it difficult to compare findings or aggregate evidence. Recognizing the need for standardized terminology to facilitate both research and clinical communication, an international multidisciplinary working group convened in 2018 to develop consensus nomenclature for cognitive changes associated with anesthesia and surgery [18]. This collaborative effort resulted in the umbrella term “perioperative neurocognitive disorders” (PND), which encompasses a spectrum of cognitive impairments occurring in temporal relationship to surgical procedures.

### 3.2. Current Nomenclature Framework

The 2018 nomenclature recommendations (Table 1) define several distinct entities within the PND spectrum, differentiated primarily by timing of onset and duration [18]:

- Postoperative Delirium (POD): An acute disturbance in attention and awareness that develops in the immediate postoperative period—typically within hours to days of surgery—and fluctuates over time. Delirium diagnosis relies on clinical criteria as defined in the Diagnostic and Statistical Manual of Mental Disorders, Fifth Edition (DSM-5) [19]. The DSM-5 requires: (1) disturbance in attention and awareness, (2) development over a short period with fluctuation in severity, (3) additional disturbance in cognition, (4) disturbances not better explained by pre-existing neurocognitive disorder, and (5) evidence that the disturbance results from a medical condition, substance, or multiple etiologies.

Delirium is further classified into three motor subtypes: hyperactive (characterized by agitation, restlessness, and psychomotor activation), hypoactive (marked by lethargy, decreased motor activity, and withdrawal), and mixed (alternating between hyperactive and hypoactive features) [20]. The hypoactive subtype is particularly prone to under-recognition in clinical practice despite being associated with worse outcomes.

- Delayed Neurocognitive Recovery: Cognitive decline identified within 30 days of surgery—but beyond the immediate acute delirium timeframe—through formal assessment. This category captures patients who demonstrate measurable cognitive impairment in the early postoperative period but do not meet criteria for delirium.
- Postoperative Neurocognitive Disorder (postoperative NCD): Cognitive decline persisting up to 12 months after surgery, identified through objective assessment relative to preoperative baseline. This term replaces the previous designation “POCD” and aligns with broader neurocognitive disorder nomenclature.

The nomenclature framework intentionally avoids specifying beyond 12 months, recognizing that attribution of cognitive changes to surgical or anesthetic factors becomes increasingly difficult with longer intervals. Other factors, including underlying neurodegenerative processes, may predominate over time.

### 3.3. Relationship to Established Delirium and Neurocognitive Disorder Classifications

The PND nomenclature intentionally aligns with established diagnostic systems, particularly the DSM-5 [19]. Postoperative delirium meets standard DSM-5 criteria for delirium, with surgery and anesthesia representing the precipitating medical condition. This alignment facilitates application of validated delirium assessment tools and ensures consistency with terminology used in psychiatric and geriatric medicine. For postoperative neurocognitive disorder, the nomenclature draws conceptually from DSM-5 neurocognitive disorder criteria, though it recognizes important differences in assessment approach. Specifically, while DSM-5 neurocognitive disorder diagnosis relies primarily on clinical judgment regarding functional impairment, research definitions of postoperative NCD have typically employed neuropsychological testing with statistical thresholds for decline. Reconciling these different approaches remains an area of ongoing discussion in the field.

### 3.4. Clinical and Research Implications

The standardized PND nomenclature serves several important functions. For clinical practice, it provides clear terminology for documenting and communicating about perioperative cognitive changes, potentially improving recognition and management. For research, standardized definitions facilitate cross-study comparisons, meta-analyses, and collaborative investigations. For patients and families, clearer terminology may enhance understanding and help set appropriate expectations regarding potential cognitive changes associated with surgery. However, important challenges remain. The distinction between delayed neurocognitive recovery and postoperative NCD based solely on timing—before versus after 30 days—may be somewhat arbitrary. Optimal assessment approaches and diagnostic thresholds continue to be debated. Additionally, translation of research definitions into practical clinical diagnostic criteria remains incomplete. As the field continues to evolve, further refinement of nomenclature and diagnostic approaches will likely be necessary to optimize clinical utility while maintaining scientific rigor. Integration with emerging biomarker research and neuroimaging findings may ultimately enable more mechanistically informed classification systems that better predict outcomes and guide targeted interventions.

## 4. Epidemiology and Clinical Significance

### 4.1. Incidence and Prevalence

The reported incidence and prevalence of perioperative neurocognitive disorders (PNDs) vary substantially because studies differ in case definitions (e.g., delirium vs. POCD), screening instruments (e.g., CAM/CAM-ICU/4AT vs. chart review), and ascertainment windows (PACU, ward days 1–3, ICU days 1–7, or post-discharge), making pooled “headline” ranges difficult to apply to individual patients [17,18,21,22,23]. Delirium risk is best interpreted after stratification by baseline vulnerability and perioperative trajectory (Table 2). In general surgical ward populations, postoperative delirium is commonly reported around 10–15%, but incidence rises sharply in high-risk strata, including older adults with frailty or pre-existing cognitive impairment and those exposed to emergency surgery or critical illness [17,24,25,26,27]. Procedure type further differentiates risk: after cardiac surgery, delirium incidence is frequently reported in the ~25–50% range and is influenced by patient age and perioperative complexity [19,28], whereas urgent orthopaedic surgery, particularly hip fracture repair in older adults, represents one of the highest-risk groups, with delirium often affecting approximately one-half of patients in vulnerable cohorts [7,27,29,30]. Care setting is also a major modifier; ICU populations, especially mechanically ventilated patients, experience a substantially higher delirium burden than non-ICU cohorts [31,32]. POCD follows a different temporal pattern and therefore should be reported by timepoint: in the ISPOCD1 study of elderly patients undergoing major non-cardiac surgery, POCD was observed in ~25% at one week and ~10% at three months, with longer-term follow-up studies suggesting persistence in a subset depending on definitions and methodology [16,20,33,34]. Given increasing global surgical volume and population aging, the absolute burden of PND is expected to rise, with the greatest concentration of cases occurring in these high-risk subgroups [1,4,17].

### 4.2. Clinical Outcomes and Long-Term Consequences

The immediate clinical consequences of postoperative delirium are well documented. These include prolonged hospital stays, increased rates of postoperative complications, and elevated risk of discharge to institutional care rather than home [7,29]. Patients who develop delirium also face substantially higher short-term mortality, with hazard ratios ranging from 1.5 to 2.0 depending on the population studied and the duration of follow-up [30]. Perhaps more concerning than these immediate outcomes is the emerging evidence of long-term cognitive consequences. Multiple longitudinal studies have now documented associations between postoperative delirium and subsequent cognitive decline [6,24]. In a seminal study, Saczynski and colleagues demonstrated that elderly patients who developed delirium after major surgery experienced significantly steeper cognitive decline over subsequent months compared to those who remained delirium-free, even after adjusting for baseline cognitive status and other potential confounders [6]. The relationship between delirium and dementia has become an area of intense investigation. Multiple population-based cohort studies have identified delirium as an independent risk factor for subsequent dementia diagnosis [8,9,36]. Recent large-scale real-world evidence from a retrospective cohort study of 164,520 hospital patients in New South Wales, Australia, demonstrated that delirium substantially increases the risk of incident dementia. Among patients without prior dementia, those experiencing delirium had significantly higher dementia incidence (adjusted hazard ratio 2.17, 95% CI 2.04–2.31) compared to those who remained delirium-free. This association persisted across age groups and was particularly pronounced in patients with subsyndromal cognitive impairment at baseline [37]. Whether delirium represents primarily a marker of underlying brain vulnerability, directly accelerates neurodegenerative processes, or involves elements of both remains under investigation. Regardless of the precise mechanistic relationship, this association carries profound implications for long-term patient outcomes and reinforces the critical importance of delirium prevention.

### 4.3. Healthcare Utilization and Economic Impact

The economic burden of PNDs is substantial and extends across multiple domains of healthcare utilization. In the United States alone, delirium-associated healthcare costs have been estimated to exceed USD 164 billion annually [5]. These costs arise from prolonged hospitalization, increased nursing care requirements, additional diagnostic testing, management of complications, and post-acute care needs including rehabilitation and long-term institutional placement [31]. Individual patient costs are considerable. Delirium adds an estimated USD 16,000 to USD 64,000 to hospital costs per patient, depending on the care setting and patient population [38]. For intensive care unit patients specifically, the presence of delirium has been associated with increased hospital costs exceeding USD 40,000 per patient, driven primarily by extended ICU and hospital lengths of stay [32]. Beyond direct medical costs, PND generates substantial indirect costs through caregiver burden, lost productivity, and long-term care needs. Patients who develop postoperative delirium face increased rates of discharge to skilled nursing facilities rather than home, with associated long-term care expenses. For those who do return home, persistent cognitive impairment may necessitate informal caregiving by family members, creating additional economic and social burdens that extend well beyond the healthcare system. The cost-effectiveness of PND prevention strategies represents an important consideration for healthcare systems. Economic analyses of multicomponent delirium prevention interventions suggest these programs can be cost-neutral or even cost-saving despite requiring additional resources for implementation. The savings come primarily through reductions in hospital length of stay and downstream care needs [39]. However, broader implementation remains limited by organizational challenges, staffing constraints, and reimbursement barriers. From a public health perspective, the aging global population and increasing surgical volumes ensure that PND will consume growing healthcare resources in coming decades unless effective prevention and management strategies are more widely adopted. This reality underscores the urgency of translating research findings into routine clinical practice and developing novel interventions to reduce PND incidence and mitigate its consequences.

## 5. Pathophysiology

### 5.1. Overview of Mechanisms

The pathophysiology of perioperative neurocognitive disorders involves a complex, multifactorial process with interactions between patient vulnerability factors, surgical trauma, anesthetic agents, and physiologic perturbations [40]. Rather than a single causative mechanism, current evidence supports a model in which multiple pathways converge to disrupt normal neuronal function and connectivity, manifesting clinically as delirium or cognitive dysfunction. Several core mechanisms have been implicated: neuroinflammation, neurotransmitter imbalances, impaired cerebral metabolism and perfusion, oxidative stress, and blood–brain barrier dysfunction [41]. These processes likely interact dynamically. Inflammation can trigger neurotransmitter dysregulation, metabolic disturbances may exacerbate oxidative stress, and blood–brain barrier compromise can facilitate peripheral immune signaling to the central nervous system. The concept of brain vulnerability is central to understanding PND pathophysiology. Patients with reduced cognitive reserve—whether from aging, pre-existing neurodegenerative disease, cerebrovascular disease, or other factors—demonstrate increased susceptibility to the same precipitating insults [42]. This vulnerability-stress model helps explain why PND incidence varies dramatically across patient populations despite similar surgical exposures.

### 5.2. Neuroinflammation

Neuroinflammation has emerged as a central mechanism in PND pathogenesis, supported by extensive preclinical research and growing human evidence [43]. Surgery triggers a systemic inflammatory response characterized by release of pro-inflammatory cytokines including interleukin-1β (IL-1β), interleukin-6 (IL-6), and tumor necrosis factor-α (TNF-α) [44]. These peripheral inflammatory signals can communicate with the central nervous system through multiple routes: circumventricular organs lacking an intact blood–brain barrier, vagal nerve signaling, and active transport across the blood–brain barrier [41]. Within the brain, peripheral inflammatory signals activate microglia—the resident immune cells of the central nervous system. Activated microglia adopt a pro-inflammatory phenotype, releasing additional cytokines, chemokines, and other inflammatory mediators. This microglial activation can persist beyond resolution of the initial peripheral inflammatory stimulus, potentially contributing to prolonged cognitive impairment [43]. Human studies have documented associations between perioperative inflammatory markers and PND risk. Patients who develop postoperative delirium demonstrate elevated levels of pro-inflammatory cytokines including IL-6, IL-8, and C-reactive protein compared to patients who remain delirium-free [44,45]. Notably, some studies suggest that pre-existing elevated inflammatory markers may predict subsequent delirium risk, consistent with the concept of pre-existing brain vulnerability [46]. The neuroinflammation hypothesis is further supported by preclinical models demonstrating that surgical trauma triggers microglial activation, blood–brain barrier disruption, and cognitive deficits in rodents [43]. Interventions targeting inflammatory pathways in these models can prevent or attenuate cognitive dysfunction, supporting a causal role for neuroinflammation. However, translating anti-inflammatory strategies to human PND prevention has proven challenging, with limited success to date.

### 5.3. Neurotransmitter Dysregulation

Abnormalities in neurotransmitter systems, particularly acetylcholine and dopamine, have long been implicated in delirium pathogenesis [47]. The cholinergic deficiency hypothesis posits that reduced cholinergic neurotransmission contributes to the attention and awareness disturbances characteristic of delirium. Supporting evidence includes the observation that anticholinergic medications are well-established delirium risk factors, and that conditions associated with cholinergic deficits (such as dementia) increase delirium susceptibility. Conversely, excess dopaminergic activity has been proposed to contribute to delirium, particularly hyperactive manifestations. This hypothesis draws support from observations that dopaminergic medications can precipitate delirium, while dopamine antagonists (antipsychotics) have been employed for symptom management—though without clear evidence of preventing delirium onset. More recent formulations recognize that multiple neurotransmitter systems are likely involved, including γ-aminobutyric acid (GABA), glutamate, serotonin, and norepinephrine [47]. Anesthetic agents directly modulate several of these systems, potentially contributing to acute postoperative cognitive changes. The complex interactions between neurotransmitter systems and their relationship to specific clinical manifestations of PND remain active areas of investigation.

### 5.4. Cerebral Perfusion and Metabolism

Adequate cerebral perfusion and metabolic substrate delivery are essential for normal neuronal function. Perioperative hypotension, hypoxemia, anemia, and impaired cerebral autoregulation can all compromise brain oxygen and glucose delivery, potentially contributing to PND [48]. Studies have documented associations between intraoperative hypotension and increased postoperative delirium risk, though optimal blood pressure targets and the impact of blood pressure variability remain subjects of debate [48]. Cerebral metabolic rate decreases under general anesthesia, but the relationship between anesthetic depth, cerebral metabolism, and PND risk is complex. While some studies suggest associations between deeper anesthesia (as measured by processed EEG monitoring) and increased delirium or cognitive dysfunction [49,50], others have not confirmed these relationships. The optimal approach to anesthetic depth management for PND prevention remains an active research question. Age-related changes in cerebral vasculature and autoregulation may increase vulnerability to perfusion-related insults. Elderly patients demonstrate reduced cerebrovascular reserve and impaired ability to maintain cerebral perfusion across varying systemic blood pressures, potentially explaining their increased PND susceptibility.

### 5.5. Blood–Brain Barrier Dysfunction

The blood–brain barrier (BBB) normally restricts passage of circulating cells, proteins, and other molecules into the brain parenchyma. Emerging evidence suggests that surgery and systemic inflammation can compromise BBB integrity, facilitating entry of peripheral immune cells and inflammatory mediators into the central nervous system [51]. Human studies have identified biomarkers of endothelial activation and BBB injury in patients who develop postoperative delirium [51]. Preclinical models demonstrate that surgical trauma increases BBB permeability, allowing peripheral immune cell infiltration and contributing to neuroinflammation and cognitive deficits [43]. Interventions that preserve BBB integrity in animal models can prevent surgery-induced cognitive dysfunction, supporting a causal role for barrier disruption. Age-related BBB changes—including increased baseline permeability and reduced repair capacity—may contribute to elderly patients’ increased PND vulnerability. Additionally, conditions such as hypertension, diabetes, and cerebrovascular disease that affect vascular health may further compromise BBB function and increase susceptibility to perioperative insults.

### 5.6. Oxidative Stress and Cellular Dysfunction

Surgical trauma and systemic inflammation generate reactive oxygen species (ROS) and reactive nitrogen species that can damage cellular components including lipids, proteins, and DNA. The brain is particularly vulnerable to oxidative stress due to its high metabolic rate, abundant lipid content, and relatively limited antioxidant defenses. Oxidative stress can impair mitochondrial function, disrupt synaptic transmission, and trigger neuronal apoptosis. While direct human evidence linking perioperative oxidative stress to PND remains limited, preclinical studies demonstrate increased oxidative markers following surgery and anesthesia, with associations to cognitive dysfunction. Antioxidant interventions in animal models have shown promise in preventing surgery-induced cognitive changes, though translation to human studies has been limited thus far.

### 5.7. Integration and Clinical Implications

The emerging picture of PND pathophysiology involves multiple interacting mechanisms rather than a single causative pathway. Surgical trauma triggers systemic inflammation, which communicates with the brain through various routes—including a compromised blood–brain barrier—activating microglia and initiating neuroinflammatory cascades. These processes disrupt neurotransmitter systems, impair cerebral metabolism, and generate oxidative stress, ultimately manifesting as clinical delirium or cognitive dysfunction. Patient-specific vulnerability factors—including age, pre-existing cognitive impairment, genetic susceptibility, and comorbid conditions—modulate individual responses to these insults, explaining the heterogeneity in PND risk. Understanding these mechanistic pathways offers potential targets for therapeutic intervention, though translating this knowledge into effective preventive or treatment strategies remains challenging. Future research integrating clinical phenotyping with mechanistic biomarkers, neuroimaging, and genetic studies will be essential to fully elucidate PND pathophysiology and identify novel therapeutic approaches. The ultimate goal is to move toward personalized risk stratification and targeted interventions based on individual mechanistic profiles.

## 6. Risk Factors and Assessment Tools

### 6.1. Risk Factor Categories

Risk factors for perioperative neurocognitive disorders can be organized conceptually into three broad categories (Table 3): predisposing (patient-related) factors, precipitating (surgery/anesthesia-related) factors, and modifiable perioperative factors [24]. Understanding these risk domains is essential for developing targeted prevention strategies and conducting individualized risk assessments.

- Predisposing factors reflect baseline patient vulnerability. These include advanced age (particularly ≥70 years), pre-existing cognitive impairment or dementia, history of prior delirium, depression, sensory impairment (vision and hearing deficits), functional dependence, comorbidity burden, frailty, alcohol use disorder, and potentially genetic susceptibility factors [24,42]. Among these, age and baseline cognitive status emerge as the most consistent and robust predictors across studies.
- Precipitating factors relate to the surgical procedure and perioperative course. These include emergency surgery (versus elective), surgical complexity and duration, specific procedure types (with cardiac surgery, vascular surgery, and hip fracture repair conferring particularly high risk), intraoperative hypotension or hypoxemia, blood loss and transfusion requirements, metabolic derangements, infection, and pain [19,25]. The magnitude of surgical stress and physiologic perturbation generally correlates with PND risk.

A recent individual patient data meta-analysis of 9384 noncardiac surgery patients identified several modifiable perioperative factors significantly associated with postoperative delirium. Independent predictors included preoperative benzodiazepine use (OR 1.62, 95% CI 1.33–1.97), intraoperative hypotension (mean arterial pressure < 65 mmHg for >10 min; OR 1.31, 95% CI 1.09–1.58), postoperative pain inadequately controlled (OR 1.89, 95% CI 1.45–2.47), and prolonged surgical duration (OR 1.21 per hour, 95% CI 1.12–1.31). Importantly, this analysis demonstrated that combinations of risk factors showed synergistic rather than simply additive effects on delirium risk [52].

- Modifiable perioperative factors represent potential intervention targets. These include medications (particularly anticholinergic agents, benzodiazepines, and certain analgesics), sleep deprivation, immobilization, dehydration, use of physical restraints, bladder catheterization, sensory deprivation (lack of eyeglasses or hearing aids), and environmental factors [24]. These factors are particularly important because they can be addressed through systematic prevention protocols.

The predisposing-precipitating model, originally developed by Inouye and Charpentier [27], posits that PND risk results from interactions between baseline vulnerability and acute precipitating insults. Patients with high baseline vulnerability—such as elderly individuals with pre-existing cognitive impairment—may develop delirium with relatively minor precipitating factors. Conversely, patients with low baseline vulnerability require more severe insults to develop delirium. This framework helps explain individual variation in PND susceptibility and guides risk-stratified intervention approaches.

### 6.2. Validated Risk Prediction Models

Several validated prediction models have been developed to estimate individual patient PND risk based on pre-identified risk factors. These tools facilitate systematic risk assessment and can guide targeted prevention efforts. For cardiac surgery patients, Rudolph and colleagues developed and validated a delirium prediction rule incorporating age, Mini-Mental State Examination (MMSE) score, history of stroke, and abnormal albumin level [53]. This model demonstrated good discrimination (area under the curve 0.77) and has been externally validated in multiple cohorts. In non-cardiac surgical populations, various prediction models have been proposed, though external validation has been more limited. The AWOL tool (Age, inability to spell “World” backwards, disOrientation, and iLlness severity) was derived and validated for predicting delirium in hospitalized medical patients and has shown promise in surgical populations as well [54]. More recently, machine learning approaches leveraging electronic health record data have been developed to predict delirium risk [55]. These models can incorporate numerous variables—including demographics, comorbidities, medications, laboratory values, and vital signs—to generate individualized risk estimates. While potentially more accurate than simpler scoring systems, these approaches require sophisticated informatics infrastructure and have not yet been widely implemented in clinical practice. Frailty assessment has emerged as a particularly valuable approach for identifying vulnerable surgical patients at high PND risk [56]. Various frailty measures, including both performance-based assessments (such as gait speed) and deficit accumulation indices, predict postoperative complications including delirium and cognitive dysfunction. Integration of frailty assessment into preoperative evaluation represents an important opportunity for improved risk stratification.

### 6.3. Clinical Assessment Tools for Delirium Detection

Systematic delirium assessment using validated instruments is essential for recognition, given that clinical judgment alone fails to detect the majority of cases [10]. Multiple tools have been developed and validated for delirium detection in various clinical settings (Table 4). The Confusion Assessment Method (CAM) [21] remains the most widely used and extensively validated delirium assessment tool. The CAM diagnostic algorithm requires: (1) acute onset and fluctuating course, (2) inattention, and (3) either disorganized thinking or altered level of consciousness. The CAM demonstrates high sensitivity (94–100%) and specificity (90–95%) when administered by trained assessors, though performance varies with user experience and training. For intensive care unit patients, the Confusion Assessment Method for the ICU (CAM-ICU) [22] adapts the CAM framework for use with mechanically ventilated and nonverbal patients. The CAM-ICU employs nonverbal attention assessments and observational items to enable delirium detection even in patients unable to communicate verbally. Extensive validation studies support its reliability and validity across diverse ICU populations. The 4AT (Assessment Test for Delirium and Cognitive Impairment) [23] represents a newer, ultra-brief screening instrument requiring less than 2 min to administer. The 4AT assesses alertness, orientation, attention (months backwards), and acute change or fluctuation, generating a score from 0 to 12. Scores ≥ 4 suggest possible delirium or cognitive impairment warranting further evaluation. The 4AT’s brevity and ease of use make it particularly attractive for busy clinical settings, and validation studies demonstrate good sensitivity and specificity. Additional tools include the Delirium Rating Scale-Revised-98 (DRS-R-98), which provides more detailed symptom assessment useful for research studies and tracking symptom severity over time, and the Nursing Delirium Screening Scale (Nu-DESC), designed for rapid bedside screening by nurses.

### 6.4. Cognitive Assessment Approaches

Assessment of delayed neurocognitive recovery and postoperative neurocognitive disorder requires formal cognitive testing, as these entities are defined by measurable decline from preoperative baseline [18]. Various approaches have been employed in research studies, though optimal methods for clinical practice remain debated. Comprehensive neuropsychological test batteries—assessing multiple cognitive domains including attention, memory, executive function, and processing speed—represent the gold standard for detecting and characterizing cognitive dysfunction [16]. However, these assessments require specialized expertise and substantial time (typically 1–2 h), making them impractical for routine clinical screening. Brief cognitive screening instruments offer more practical alternatives for clinical settings. The Montreal Cognitive Assessment (MoCA) [57] has gained widespread use as a sensitive screening tool for mild cognitive impairment. The MoCA assesses multiple cognitive domains in approximately 10 min and demonstrates superior sensitivity compared to the older Mini-Mental State Examination (MMSE) for detecting mild cognitive changes. Preoperative MoCA assessment can establish baseline cognitive status and be repeated postoperatively to detect decline. Computerized cognitive testing platforms offer standardized, automated assessment that can be administered by non-specialist personnel. These tools typically assess reaction time, attention, memory, and executive function through brief computerized tasks. While offering advantages in standardization and ease of administration, questions remain regarding optimal change thresholds and clinical interpretation. A fundamental challenge in defining cognitive decline is determining the threshold for clinically significant change. Statistical approaches have employed various criteria—including decline of 1 or 2 standard deviations from baseline on individual tests, or decline on a specified proportion of tests in a battery. However, these statistical thresholds may not correspond to meaningful functional impairment. Integration of functional status assessment with cognitive testing represents an important direction for future work.

### 6.5. Biomarker Research

Investigation of biomarkers for PND risk prediction, diagnosis, and prognosis represents an active research frontier. Various candidate biomarkers have been explored, including inflammatory markers, neuronal injury markers, genetic variants, and neuroimaging findings [58]. Inflammatory biomarkers—particularly IL-6, IL-8, C-reactive protein, and S100β—have shown associations with delirium in multiple studies [45]. However, specificity remains limited, as these markers are elevated in many acute illness states. Whether inflammatory biomarkers add predictive value beyond clinical risk assessment remains uncertain. Cerebrospinal fluid (CSF) biomarkers including neurofilament light chain, tau proteins, and amyloid-β have been investigated as potential indicators of neuronal injury and delirium risk [58]. While some studies document associations between CSF biomarkers and delirium, the invasive nature of CSF sampling limits clinical applicability. Blood-based versions of these neuronal injury markers are under investigation and may offer more practical alternatives. Genetic studies have identified several candidate susceptibility genes, including those involved in inflammation (e.g., IL-6, TNF-α), neurotransmission (e.g., cholinergic and dopaminergic pathway genes), and apolipoprotein E (APOE) [59]. However, effect sizes are generally modest, and clinical genetic testing for PND risk assessment is not currently recommended. Neuroimaging research has documented associations between brain structure—such as cortical atrophy and white matter hyperintensities—and delirium susceptibility [59]. Advanced techniques including diffusion tensor imaging and functional connectivity MRI may provide insights into neural network vulnerabilities predisposing to PND. However, routine preoperative neuroimaging for PND risk assessment is not supported by current evidence. Integration of multiple biomarker modalities into comprehensive risk prediction models represents a promising future direction. However, substantial work remains to identify biomarkers with sufficient predictive value to justify clinical implementation and cost.

## 7. Prevention Strategies

### 7.1. Multicomponent Non-Pharmacological Interventions

Multicomponent non-pharmacological interventions that target multiple delirium risk factors simultaneously represent the most effective evidence-based approach to PND prevention [12,13]. These programs systematically address modifiable risk factors including cognitive impairment, sleep deprivation, immobility, dehydration, vision and hearing impairment, and inappropriate medications. The Hospital Elder Life Program (HELP), developed by Inouye and colleagues, represents the prototype and most extensively studied multicomponent intervention (Table 5) [60,61]. HELP employs trained volunteers and interdisciplinary staff to deliver standardized protocols addressing six risk factors: cognitive impairment (through daily orientation and therapeutic activities), sleep deprivation (nonpharmacologic sleep enhancement protocol), immobility (early mobilization and range-of-motion exercises), vision impairment (ensuring availability of eyeglasses and adaptive equipment), hearing impairment (ensuring hearing aids and communication strategies), and dehydration (encouraging oral fluid intake). In the original randomized controlled trial, HELP reduced delirium incidence by 40% (9.9% vs. 15.0%, *p* = 0.02) without increasing costs [62]. Subsequent studies across diverse healthcare settings have demonstrated consistent efficacy, with meta-analyses confirming approximately 30–40% relative risk reduction for delirium [12]. Additionally, HELP implementation has been associated with reduced falls, decreased use of physical restraints and psychoactive medications, and lower overall healthcare costs [63]. Adaptations of the HELP model for surgical populations have demonstrated similar efficacy. Chen and colleagues implemented a modified HELP program for abdominal surgery patients, demonstrating reduced delirium incidence (12.5% vs. 24.2%, *p* = 0.03) and shorter hospital length of stay [26].

Other studies have successfully adapted multicomponent interventions for cardiac surgery, orthopedic surgery, and intensive care unit settings. Key elements contributing to multicomponent intervention success include: systematic risk screening to identify vulnerable patients; standardized protocols for risk factor modification; involvement of trained staff and volunteers; family engagement and education; and interdisciplinary coordination. Successful implementation requires institutional commitment, staff training, and often cultural change in care delivery. Despite strong evidence supporting multicomponent interventions, implementation remains limited across healthcare systems. Barriers include resource requirements (staff time, volunteer coordination), competing clinical priorities, lack of reimbursement mechanisms, and organizational culture. The 2024 update of the European Society of Anaesthesiology and Intensive Care Medicine evidence-based guidelines provides Level A (strong) recommendations for implementing multicomponent non-pharmacological interventions as first-line prevention in all at-risk surgical patients, emphasizing that such programs should be considered a fundamental component of perioperative care pathways rather than optional add-ons [27]. Strategies to enhance dissemination and implementation represent important priorities for translating evidence into practice.

### 7.2. Pharmacological Prevention

Despite extensive investigation, no pharmacological agent has demonstrated consistent efficacy for delirium prevention with an acceptable safety profile, and current guidelines do not recommend routine pharmacological prophylaxis (Table 6) [64]. Specifically:

- Antipsychotics: Prophylactic administration of antipsychotic medications—including haloperidol, risperidone, and olanzapine—has been evaluated in multiple randomized trials. While some early studies suggested potential benefits, larger and more rigorous trials have generally failed to demonstrate reduced delirium incidence [65,66]. A 2016 Cochrane systematic review concluded that antipsychotics do not prevent delirium and may cause harm through adverse effects including extrapyramidal symptoms and oversedation [13]. Current guidelines recommend against routine antipsychotic prophylaxis.
- Cholinesterase Inhibitors: Given the cholinergic deficiency hypothesis of delirium, cholinesterase inhibitors (donepezil, rivastigmine) have been investigated for prevention. However, randomized trials have consistently failed to demonstrate efficacy and have raised safety concerns including increased bradycardia risk [67]. These agents are not recommended for delirium prevention.
- Alpha-2 Agonists: Dexmedetomidine, an alpha-2 adrenergic agonist with sedative and analgesic properties, has shown promise in some studies. A large randomized trial in elderly non-cardiac surgery patients demonstrated that prophylactic low-dose dexmedetomidine reduced delirium incidence (9% vs. 23%, *p* < 0.001) [68]. However, questions regarding optimal dosing, patient selection, timing, and safety—particularly cardiovascular effects—remain.

A subsequent randomized controlled trial (the MINDDS study) specifically investigated nighttime dexmedetomidine infusion (0.1 μg/kg/h from 21:00 to 06:00 for three consecutive nights) in non-mechanically ventilated patients following cardiac surgery. This circadian-aligned approach demonstrated significant reduction in delirium incidence (4.8% vs. 16.7%; dexmedetomidine vs. placebo, *p* = 0.008) without increasing sedation-related adverse events. The authors proposed that targeting the sleep–wake cycle disruption specifically during nighttime hours may optimize dexmedetomidine’s preventive effects while minimizing daytime sedation [69]. A comprehensive Bayesian network meta-analysis of 41 randomized trials (8218 cardiac surgery patients) evaluated multiple perioperative pharmacological interventions. The analysis confirmed dexmedetomidine’s superiority over placebo (RR 0.41, 95% credible interval 0.28–0.61) and demonstrated it had the highest probability (78%) of being the most effective intervention for delirium prevention. Importantly, the network meta-analysis allowed indirect comparisons showing dexmedetomidine’s superiority over haloperidol (RR 0.52, 95% CrI 0.31–0.87) and comparable efficacy to multicomponent interventions when both were compared to standard care [70]. Further research is needed before routine prophylactic use can be recommended. Other alpha-2 agonists including clonidine have shown mixed results in smaller studies [71].

- Melatonin and Melatonin Receptor Agonists: Ramelteon, a melatonin receptor agonist, demonstrated delirium prevention efficacy in a Japanese randomized trial [28]. However, replication in other populations has been limited. Melatonin itself has shown promise in some observational studies but lacks definitive randomized controlled trial evidence. These agents warrant further investigation but are not currently recommended for routine prophylaxis.
- Other Agents: Various other pharmacological approaches—including antioxidants, anti-inflammatory agents, and neuroprotective compounds—have been investigated in preclinical models or small clinical studies, but robust evidence supporting clinical use is lacking.

The limited success of pharmacological prevention efforts may reflect the multifactorial nature of PND pathophysiology, suggesting that single-agent interventions are unlikely to provide comprehensive protection. Future research might focus on combination pharmacological approaches targeting multiple mechanisms, or personalized pharmacological interventions based on individual mechanistic profiles.

### 7.3. Anesthetic Management Strategies

Anesthetic technique, agent selection, and depth of anesthesia represent potentially modifiable factors that may influence PND risk, though optimal approaches remain debated.

- Anesthetic Depth Monitoring: The hypothesis that deeper anesthesia increases neurocognitive risk has led to investigation of processed electroencephalogram (EEG) monitoring to guide anesthetic titration. Several randomized trials have evaluated whether maintaining lighter anesthesia—based on bispectral index or other EEG measures—reduces delirium or cognitive dysfunction compared to routine care [44,72].

Results have been mixed. Some studies demonstrated reduced delirium with EEG-guided anesthesia [49,50], while others showed no benefit [72]. A meta-analysis suggested modest benefit for delirium prevention but no clear impact on longer-term cognitive outcomes. Current evidence does not definitively support routine EEG monitoring for PND prevention, though avoiding unnecessarily deep anesthesia appears prudent.

- Regional Anesthesia: The hypothesis that regional anesthesia (spinal, epidural, or peripheral nerve blocks) might reduce PND risk compared to general anesthesia has been extensively investigated. The proposed mechanisms include avoiding general anesthetic agents’ direct neurotoxic effects, reducing perioperative opioid requirements, and enabling earlier mobilization.

However, randomized trials comparing regional versus general anesthesia for hip fracture surgery and other procedures have generally failed to demonstrate significant differences in delirium incidence [73,74]. A Cochrane systematic review concluded that evidence does not support superiority of either technique for delirium prevention. Regional anesthesia may offer other benefits—such as reduced pulmonary complications and improved pain control—but does not appear to substantially alter delirium risk when used alone.

- Anesthetic Agent Selection: Whether choice of specific anesthetic agents influences PND risk remains uncertain. Some observational studies have suggested associations between volatile anesthetic exposure and increased cognitive dysfunction risk, while propofol-based total intravenous anesthesia (TIVA) has been proposed as potentially neuroprotective. However, randomized trials have not consistently demonstrated differences in neurocognitive outcomes between volatile agents and TIVA.

Similarly, comparisons between different volatile agents (sevoflurane, desflurane, isoflurane) have not revealed clear differences in PND risk. Current evidence does not support selecting specific anesthetic agents primarily for PND prevention.

- Sedation Depth in Non-General Anesthesia Cases: For procedures performed under spinal anesthesia or monitored anesthesia care, sedation depth may influence delirium risk. The STRIDE randomized trial demonstrated that lighter sedation—targeting minimal to moderate sedation—during hip fracture repair under spinal anesthesia reduced delirium incidence compared to deeper sedation (13.4% vs. 22.1%, *p* = 0.04) [72]. This finding supports minimizing sedation depth when possible, particularly in high-risk patients.

### 7.4. Optimization of Perioperative Care Pathways

Integration of PND prevention into comprehensive perioperative care pathways offers opportunities to address multiple risk factors systematically:

- Enhanced Recovery After Surgery (ERAS) Protocols: ERAS pathways incorporate evidence-based interventions across the perioperative continuum, including preoperative patient optimization, minimally invasive surgical techniques, optimized anesthesia and analgesia, early mobilization, and early oral nutrition. While ERAS protocols were not designed specifically for PND prevention, several components directly address delirium risk factors. Studies evaluating ERAS implementation have demonstrated reduced delirium rates in some surgical populations, though specific effects are difficult to isolate from overall protocol benefits.
- Comprehensive Geriatric Assessment and Co-Management: Proactive geriatric consultation and co-management for high-risk elderly surgical patients represents another promising approach. Geriatricians can optimize medical management, address polypharmacy, identify and treat delirium promptly, and coordinate multidisciplinary care. Randomized trials of geriatric co-management for hip fracture patients have demonstrated reduced delirium duration and severity, though effects on incidence have been more variable [31].
- ICU Liberation (ABCDEF) Bundle: For critically ill surgical patients, the ICU Liberation bundle provides a systematic framework addressing multiple risk factors [75]. The bundle components include: Assess, prevent, and manage pain (A); Both spontaneous awakening and breathing trials (B); Choice of analgesia and sedation (C); Delirium assessment, prevention, and management (D); Early mobility and exercise (E); and Family engagement and empowerment (F). Implementation of the complete ABCDEF bundle has been associated with improved outcomes including reduced delirium, shorter mechanical ventilation duration, and decreased hospital mortality [75]. The bundle represents an important framework for systematic PND prevention in ICU settings, though implementation challenges remain substantial.

## 8. Management of Established Delirium

### 8.1. Recognition and Diagnostic Evaluation

Prompt recognition of delirium when it occurs is essential for appropriate management and investigation of underlying causes. As noted previously, systematic screening using validated tools (CAM, CAM-ICU, 4AT) substantially improves detection compared to routine clinical assessment alone [10,21,22,23]. Once delirium is recognized, thorough evaluation to identify and address precipitating and perpetuating factors is critical [64,76,77]. This evaluation should include:

- Comprehensive medication review: Identifying and discontinuing or minimizing deliriogenic medications including anticholinergics, benzodiazepines, antihistamines, and other psychoactive agents.
- Assessment for acute medical conditions: Systematic evaluation for infection (urinary tract infection, pneumonia, surgical site infection), metabolic derangements (hypoglycemia, hyperglycemia, electrolyte abnormalities, uremia), hypoxemia, hypotension, anemia, dehydration, urinary retention, and constipation.
- Pain assessment and management: Ensuring adequate analgesia while minimizing deliriogenic analgesics.
- Review of recent procedures and interventions: Considering iatrogenic contributions such as new medications, sleep disruption, immobilization, or sensory deprivation.

Laboratory evaluation should be tailored to clinical context but typically includes complete blood count, comprehensive metabolic panel, urinalysis, and oxygen saturation assessment. Additional studies—such as chest radiography, blood cultures, neuroimaging, or electroencephalography—should be pursued based on clinical indicators rather than routinely.

### 8.2. Non-Pharmacological Management

Non-pharmacological interventions remain the cornerstone of delirium management, with approaches similar to prevention protocols but intensified for patients with active delirium [64,77]. Current European guidelines recommend that these non-pharmacological strategies be delivered systematically through structured protocols rather than as ad hoc interventions, with regular monitoring of implementation fidelity and patient response [27].

Key interventions include:

- Reorientation: Frequent reorientation to time, place, and situation using verbal reminders, calendars, clocks, and familiar objects from home.
- Cognitive stimulation: Engaging patients in conversation, reminiscence, and therapeutic activities appropriate to their cognitive level.
- Sleep-wake cycle regulation: Minimizing nighttime disruptions, providing daytime light exposure and activity, reducing noise and unnecessary monitoring, and establishing consistent sleep–wake schedules.
- Early mobilization: Encouraging out-of-bed activity and ambulation as soon as medically appropriate, with physical therapy consultation for high-risk patients.
- Sensory optimization: Ensuring availability and use of eyeglasses, hearing aids, and dentures; adequate lighting; and communication strategies for sensory-impaired patients.
- Family involvement: Encouraging family presence and participation in reorientation and therapeutic activities, with education regarding delirium nature and management.
- Environmental modification: Providing a calm, quiet environment with consistent caregivers when possible; minimizing room transfers; and avoiding physical restraints unless absolutely necessary for safety.
- Hydration and nutrition: Ensuring adequate fluid intake and nutritional support while respecting patient preferences and abilities.

Systematic implementation of these interventions requires interdisciplinary coordination involving nurses, physicians, therapists, family members, and potentially trained volunteers. Standardized protocols and staff education enhance consistency and effectiveness.

### 8.3. Pharmacological Management

Despite widespread use of antipsychotic medications for delirium management, evidence supporting their efficacy is limited, and recent guidelines emphasize judicious use only for specific indications [64,77]. The Hope-ICU trial, a large randomized controlled trial evaluating haloperidol for delirium treatment in ICU patients, found no benefit for delirium duration, coma-free days, or mortality, and raised concerns about increased adverse effects [65]. Similarly, other trials evaluating various antipsychotics have generally failed to demonstrate efficacy for reducing delirium duration or severity. Based on this evidence, current guidelines recommend against routine antipsychotic use for delirium treatment [64,77]. Antipsychotics should be reserved for patients with severe agitation posing safety risk to themselves or others, and only after non-pharmacological interventions have been attempted and medical causes addressed. When used, the lowest effective dose should be employed for the shortest duration necessary, with regular reassessment and attempts to discontinue.

Specific medication considerations:

- Haloperidol: The most extensively studied antipsychotic for delirium, typically administered at low doses (0.5–1 mg). Caution is warranted due to QT prolongation risk and extrapyramidal effects.
- Atypical antipsychotics (risperidone, olanzapine, quetiapine): Sometimes preferred due to lower extrapyramidal symptom risk, though evidence of superiority is lacking. Metabolic and cardiovascular effects require monitoring.
- Benzodiazepines: Generally contraindicated for delirium management except in specific circumstances (alcohol or benzodiazepine withdrawal, seizures). Benzodiazepines can worsen delirium and should be avoided or minimized.
- Dexmedetomidine: May have a role in ICU settings for sedation of agitated delirious patients, particularly those requiring mechanical ventilation. Some studies suggest potential benefits over benzodiazepines or propofol, but evidence remains limited.

The primary focus of pharmacological management should remain on discontinuing deliriogenic medications rather than adding new psychoactive agents.

### 8.4. Management of Delirium Subtypes

Recognition of delirium motor subtypes (hyperactive, hypoactive, mixed) has implications for management approach [20]. Hyperactive delirium, characterized by agitation, restlessness, and combativeness, typically receives the most attention due to disruptive behaviors and safety concerns. Management emphasizes de-escalation strategies, environmental modification, family presence, and addressing underlying causes. Pharmacological intervention may be necessary when safety is compromised despite non-pharmacological approaches. Hypoactive delirium, marked by lethargy, reduced motor activity, and withdrawal, often goes unrecognized despite being associated with worse outcomes. Management focuses on early mobilization, cognitive stimulation, treatment of underlying causes, and vigilance for complications such as aspiration and pressure injuries. Sedating medications should be particularly avoided in this subtype. Mixed delirium alternates between hyperactive and hypoactive features, requiring flexible management strategies tailored to current presentation. Regardless of subtype, the fundamental management principles remain consistent: systematic assessment and treatment of underlying causes, implementation of non-pharmacological interventions, judicious use of medications when necessary, and prevention of complications.

## 9. Future Prospectives and Conclusions

Despite significant progress in understanding perioperative neurocognitive disorders, major questions remain unanswered. We need better tools to identify patients at risk and to detect delirium early, particularly the subtle hypoactive form that often goes unnoticed. The concerning link between surgical delirium and later dementia demands urgent investigation—if preventing delirium today can preserve cognitive health years later, it fundamentally changes how we should approach perioperative care. While we know multicomponent interventions work, getting them implemented consistently in busy hospitals remains challenging. We must also address troubling gaps: most research focuses on elderly patients having major surgery, leaving younger adults, children, and those having outpatient procedures understudied. Standardizing how we measure outcomes across studies would help us learn faster from each other’s work. Perhaps most importantly, we need to understand whether the burden of cognitive complications falls disproportionately on disadvantaged populations and ensure that effective prevention reaches everyone who needs it. The path forward requires not just discovering what works in research settings but figuring out how to deliver that care reliably to all patients.

## Figures and Tables

**Table 1:** Nomenclature of Perioperative Neurocognitive Disorders.

Term	Timing	Definition	Assessment Method
Postoperative Delirium (POD)	Acute (hours to days after surgery)	Acute disturbance in attention and awareness with fluctuating course, meeting DSM-5 criteria for delirium	Clinical assessment using validated tools (CAM, CAM-ICU, 4AT)
Delayed Neurocognitive Recovery	Up to 30 days postoperatively	Cognitive decline from preoperative baseline detected by objective assessment	Formal cognitive testing (neuropsychological battery or validated screening tools)
Postoperative Neurocognitive Disorder (Postoperative NCD)	Up to 12 months postoperatively	Persistent cognitive decline from preoperative baseline	Formal cognitive testing with documented functional impairment

Abbreviations: DSM-5, Diagnostic and Statistical Manual of Mental Disorders, Fifth Edition; CAM, Confusion Assessment Method; CAM-ICU, Confusion Assessment Method for the Intensive Care Unit; 4AT, 4 ‘A’s Test; NCD, neurocognitive disorder.

**Table 2:** Postoperative Delirium Incidence.

Surgery Type/Population	Typical Setting Where Reported	Delirium Incidence	Notes/Key Sources
General surgical patients (mixed procedures)	Non-ICU wards	~10–15%	Often cited for “general surgery,” but masks strong age/frailty effects [17].
Cardiac surgery (incl. on-pump cohorts)	ICU and step-down/ward	~25–50%	Higher-risk subgroup; perioperative complexity and ICU exposure common [33,35].
Emergency orthopaedics (esp. hip fracture in older adults)	Ward ± ICU depending on acuity	~≥50%	Particularly high in very old/frail/cognitively vulnerable patients; strong downstream impact [7,24,31,36].
Critically ill surgical patients (varied surgery types)	ICU (often mechanically ventilated)	Higher than non-ICU; wide ranges across cohorts	ICU delirium burden is consistently high, but exact percentages vary by case-mix and measurement; see ICU systematic review/meta-analysis literature [30] and cost cohort data [32].

Abbreviations: ICU, Intensive Care Unit.

**Table 3:** Risk Factors for Perioperative Neurocognitive Disorders.

Category	Risk Factors	Strength of Association
Predisposing (Patient) Factors	Advanced age (≥70 years)	Strong
	Pre-existing cognitive impairment or dementia	Strong
	History of prior delirium	Strong
	Depression	Moderate
	Sensory impairment (vision/hearing)	Moderate
	Functional dependence	Moderate
	Frailty	Strong
	Comorbidity burden	Moderate
	Alcohol use disorder	Moderate
Precipitating (Surgery/Anesthesia) Factors	Emergency surgery	Strong
	Surgical complexity and duration	Moderate
	Type of surgery (cardiac, vascular, orthopedic)	Strong
	Intraoperative hypotension	Moderate
	Blood loss/transfusion	Moderate
	Metabolic derangements	Strong
Modifiable Perioperative Factors	Anticholinergic medications	Strong
	Benzodiazepines	Strong
	Opioid analgesics	Moderate
	Sleep deprivation	Moderate
	Immobilization	Moderate
	Dehydration	Moderate
	Physical restraints	Moderate
	Bladder catheterization	Moderate
	Lack of sensory aids (glasses, hearing aids)	Moderate

Note: Strength of association based on consistency and quality of evidence across multiple studies.

**Table 4:** Validated Assessment Tools for Delirium Detection.

Tool	Setting	Administration Time	Key Features	Sensitivity	Specificity
Confusion Assessment Method (CAM)	General ward, surgical	5–10 min	Requires: (1) Acute onset/fluctuation, (2) Inattention, (3) Disorganized thinking OR altered consciousness	94–100%	90–95%
Confusion Assessment Method for the Intensive Care Unit (CAM-ICU)	Intensive care unit	2–5 min	Adapted for nonverbal patients; uses observational items and nonverbal attention tasks	75–95%	85–98%
4AT	Any acute care setting	<2 min	Ultra-brief screening: Alertness, AMT4 (orientation), Attention (months backward), Acute change	76–90%	84–93%
Nursing Delirium Screening Scale (Nu-DESC)	Any acute care setting	1–2 min	Nurse-administered observational tool; 5 items scored 0–2	85–95%	80–87%
Delirium Rating Scale-Revised-98 (DRS-R-98)	Research, detailed assessment	15–20 min	13 severity items + 3 diagnostic items; tracks symptom evolution	Not applicable (severity scale)	Not applicable

Note: Sensitivity and specificity ranges represent values reported across validation studies; performance varies with user training and clinical context.

**Table 5:** Components of Multicomponent Delirium Prevention Interventions.

Intervention Domain	Specific Interventions	Target Risk Factor
Cognitive Stimulation	Daily orientation (person, place, time, situation) Therapeutic activities (discussion, reminiscence) Cognitive games and puzzles	Cognitive impairment
Sleep Enhancement	Nighttime noise reduction Minimizing nighttime care activities Daytime light exposure Avoiding sedative-hypnotics Warm drinks, relaxation music	Sleep deprivation
Early Mobilization	Out-of-bed activity multiple times daily Ambulation or wheelchair mobility Range-of-motion exercises Physical therapy consultation	Immobility
Vision/Hearing Optimization	Ensuring the availability of eyeglasses Ensuring the availability of hearing aids Visual/hearing adaptive equipment Communication strategies	Sensory impairment
Hydration/Nutrition	Encouraging oral fluid intake Assistance with feeding Preferred foods and beverages Monitoring intake	Dehydration
Medication Review	Discontinuing deliriogenic medications Minimizing psychoactive agents Avoiding anticholinergics and benzodiazepines Pain management with minimal sedation	Medication adverse effects
Environmental Modification	Calm, quiet environment Consistent caregivers Familiar objects from home Minimizing room transfers Avoiding physical restraints	Sensory deprivation, Disorientation
Family Engagement	Liberal visitation policies Family participation in reorientation Education regarding delirium Communication strategies	Disorientation, Anxiety

Note: Components based on Hospital Elder Life Program (HELP) and similar multicomponent interventions.

**Table 6:** Evidence Summary for Pharmacological Delirium Prevention.

Drug Class	Specific Agents	Proposed Mechanism	Evidence Quality	Efficacy	Current Recommendation
Antipsychotics	Haloperidol Risperidone Olanzapine	Dopamine receptor antagonism	High (multiple RCTs, meta-analyses)	No benefit demonstrated; potential harm	Not recommended for routine prophylaxis
Cholinesterase Inhibitors	Donepezil Rivastigmine	Enhance cholinergic transmission	Moderate (several RCTs)	No benefit; safety concerns (bradycardia)	Not recommended
Alpha-2 Agonists	Dexmedetomidine Clonidine	Alpha-2 adrenergic agonism; sedative/analgesic effects	Moderate (several RCTs; mixed results)	Promising but inconsistent; questions regarding optimal dosing and safety	Further research needed; not routinely recommended
Melatonin Receptor Agonists	Ramelteon Melatonin	Sleep-wake cycle regulation	Low (limited RCTs, single population)	Some positive findings; requires replication	Further research needed; not routinely recommended
Acetaminophen	Acetaminophen	Analgesia; anti-inflammatory	Low (few RCTs)	Inconsistent findings	Insufficient evidence
Ketamine	Ketamine (sub-anesthetic doses)	NMDA receptor antagonism; analgesia	Low (observational studies)	Preliminary positive signals	Experimental; requires RCTs

Abbreviations: RCT, randomized controlled trial; NMDA, N-methyl-D-aspartate. Note: Recommendations based on current clinical practice guidelines and systematic reviews.

## Data Availability

All data generated during this study are included in this published article.
